# Selection and Application of ssDNA Aptamers for Fluorescence Biosensing Detection of Malachite Green

**DOI:** 10.3390/foods11060801

**Published:** 2022-03-10

**Authors:** Miaojia Xie, Zanlin Chen, Fengguang Zhao, Ying Lin, Suiping Zheng, Shuangyan Han

**Affiliations:** 1 Guangdong Key Laboratory of Fermentation and Enzyme Engineering, School of Biology and Biological Engineering, South China University of Technology, Guangzhou 510006, China; 201921046785@mail.scut.edu.cn (M.X.); 202121050086@mail.scut.edu.cn (Z.C.); feylin@scut.edu.cn (Y.L.); spzheng@scut.edu.cn (S.Z.); 2 School of Light Industry and Engineering, South China University of Technology, Guangzhou 510006, China; fgzhao@scut.edu.cn

**Keywords:** capture-SELEX, DNA aptamer, malachite green oxalate, fluorescent aptasensor

## Abstract

Malachite green oxalate (MG) is a kind of veterinary drug, which is freely soluble in water and hazardous to aquatic products, resulting in food toxicity and human health problems. The demand for effective and sensitive detection of MG residues is increasing in food safety. In this work, three DNA aptamers MG-36-12/16/17 targeting MG with good affinity (K_d_ values were 169.78, 71.94, and 102.46 μM, respectively) were obtained by Capture-SELEX. Furthermore, MG-36-12, MG-76-16-6A, and MG-36-17 were found to perform sensitively and specifically to detect MG as a sensing probe in a FRET fluorescent aptasensor, where the FAM-labeled aptamer and GO were employed as efficient energy donor–acceptor pair. The linear range of this aptasensor using aptamer MG-36-12 was from 1.71 to 514.29 ng/mL and the LOD was as low as 0.79 ng/mL. Additionally, the fluorescent assay using aptamer MG-36-17 to detect MG exhibited a linear relationship from 1.71 to 857.14 ng/mL and a LOD of 2.13 ng/mL. Meanwhile, the aptasensor showed high specificity to MG with no cross-reactivity to other veterinary drugs and had a mean recovery of 81.54% to 100.96% in actual water samples from the aquatic product market.

## 1. Introduction

Malachite green (MG) is a low-cost cationic triphenylmethane dye, which is also a highly effective drug to avoid fungal, bacterial, and parasitic infections. MG is often used by fishmongers to prevent infectious diseases and prolong the life of fish with damaged scales during transport and temporary farming before the sale [1]. Therefore, residues of MG and its derivatives in aquatic products and aquaculture water may cause serious food safety and the pollution of water resources. MG has well-documented ecotoxicological effects including cytotoxicity, carcinogenicity, mutagenicity, induction of chromosomal fractures, teratogenicity, and respiratory toxicity [1,2,3]. Although MG has been banned in many countries, there are still incidents of illegal use and detection of MG in aquatic products.

To ensure food quality and safety, all kinds of analytical methods are used to determine MG residues and its metabolite in aquatic products and aquatic water, such as mass spectrometry [4], liquid chromatography [5], liquid chromatography coupled with tandem mass spectrometry [6], immunological assays [7], visual and spectrophotometric determination [8], surface-enhanced Raman scattering [9], and molecularly imprinted polymer [10]. Those mentioned methods are reliable with satisfactory sensitivity, accuracy, and precision, but they also require relevant extraction pretreatment, large-scale instruments, professional experimental skills, and expensive costs [11]. Therefore, there is need for another easier, dependable, and intuitive detection method of MG to replace extant ones, making it easy to apply for the rapid detection of MG.

Aptamer is a kind of single-stranded oligonucleotides, such as single-stranded DNA (ssDNA), RNA, or other artificial nucleic acids. Aptamers can fold themselves into distinct secondary structures and further form complex dimensional structures with strong affinity and specific binding to target, including bacteria, fungi, cells, proteins, ions, small molecules, etc. The conventional method to select aptamers was established by an in vitro screening technology called the systematic evolution of ligands by exponential enrichment (SELEX) [12,13]. Grate, D. selected an RNA aptamer of MG earlier, which was supposed to be less stable compared with DNA aptamer [14]. There are little published data to date on DNA aptamers of MG except that Wu et al. reported a DNA MG aptamer A3 with μM level affinity [15].

Various aptamers acquired by SELEX are widely used in many research fields, such as molecular biology, clinical practice, medical research, and food safety [16]. For food contaminant detection, aptamers serve as a bio-recognition element for the rapid detection of food contaminant residues. While in an aptamer-based biosensor, the detection signal is required to convert into optical or electrical signals for observation. One kind of aptasensor, fluorescent sensor [17], was developed by the Förster resonance energy transfer (FRET) from the FAM-labeled aptamer and graphene oxide (GO) as efficient energy donor and acceptor, respectively. GO exhibits good water solubility and biocompatibility, providing an inexpensive platform for fluorescence-based biosensors. GO can absorb the fluorescent modified aptamer on their surface and efficiently quench the fluorescence, owing to the strong π–π stacking effect between the nucleobases of aptamer and the sp^2^ atoms of GO [18]. The fluorescent aptasensor was employed based on the relationship between the fluorescence intensity change in the system and the target concentration, which achieves easier observation and operation with an inexpensive instrument for rapid detection of MG or other food contaminations.

**Scheme 1:**
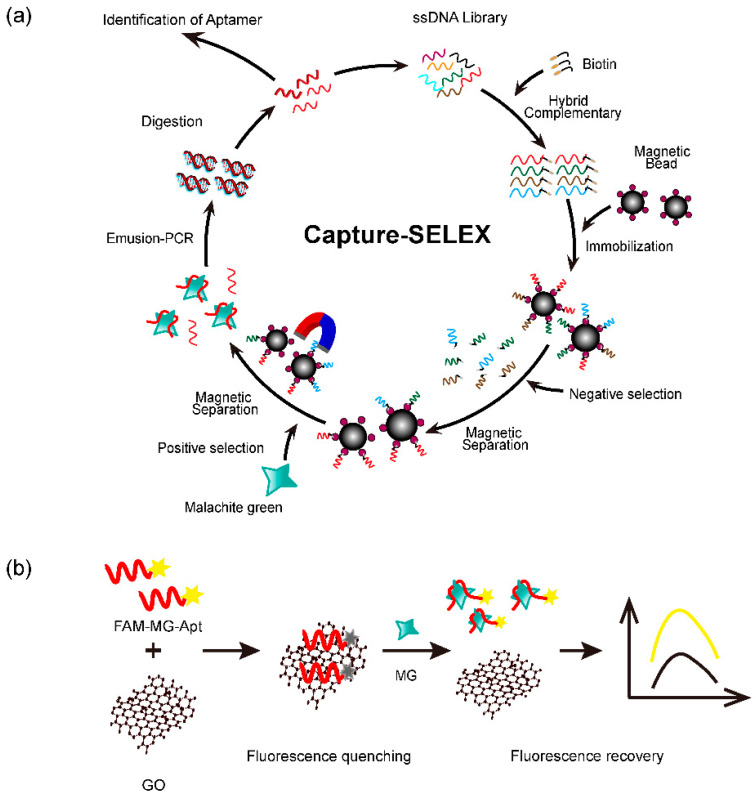
(**a**). Work procedure of library-immobilized Capture-SELEX strategy for the selection of aptamer-targeting MG. (**b**) Schematic illustration of the GO-based fluorescent aptasensor using MG aptamer probe for target detection.

In this study, our work could be succinctly summarized in two parts showing in the Scheme 1a,b, one of which was the screening of MG aptamers by Capture-SELEX, and the other was the application of aptamers in fluorescent sensor for the detection of MG. MG oxalate was used as the target in the positive selection of the SELEX process, which was one of the structural forms of MG and the most common for commercial sale. We succeeded in obtaining three DNA aptamers with good affinity and specificity for MG from an immobilized random DNA library by Capture-SELEX. These aptamers were introduced into a GO-based fluorescent aptasensor assay for sensitive and specific detection of MG. In addition, to ensure aptamers with sufficient recognition affinity, the introduction of GO also gave the sensor excellent detection capabilities [19]. This study substantially extended our knowledge of the diversity within MG aptamer sequence information. Moreover, this GO-based fluorescent aptasensor assay had grown in importance considering the recent need for a rapid, simple, accurate, and sensitive approach for detecting MG residues in fishery water samples.

## 2. Materials and Methods

### 2.1. Chemical Reagents and Materials

Malachite green oxalate (MG) standard (M_W_ 463.5, CAS:2437-29-8) was purchased from the China National Institute of Metrology (Beijing, China). Leuco malachite green (LMG, CAS:129-73-7), semicarbazide (SEM, CAS:563-41-7), 3-Amino-2-oxazolidinone (AOZ, CAS:80-65-9), 1-Aminohydantoin (AHD, CAS:2827-56-7), 5-morpholine-methyl-3-amino-2-oxazolidinone (AMOZ, CAS:43056-63-9), chloramphenicol (CAP, CAS:56-75-7), sulfamethazine (SMZ, CAS:57-68-1), crystal violet (CV, CAS:548-62-9), and sulfapyridine (SP, CAS:144-83-2) standards were purchased from Tan-Mo Technology., Ltd. (Beijing, China). The initial random ssDNA library (5′-GTTCGTGGTGTGCTGGATGT-N_36_-TGACACATCCAGCAGCACGA-3′) was synthesized by Sangon Biotech., Ltd. (Shanghai, China). The primers (Biotin-CO, FAM-Forward, PolyA_20_-Reverse, Q-Forward, and Q-Reverse, Table 1) used for immobilizing DNA library and PCR were synthesized by Nanjing Genscript Biotechnology Ltd. (Nanjing, China). 5′-FAM-MG-Aptamers were synthesized by Genewiz Biotech Co., Ltd. (Suzhou, China). The streptavidin-labeled magnetic beads were purchased from Thermo Fisher Scientific Ltd. (Shanghai, China). High-throughput sequencing was performed at the Illumine platform by Anhui Angputuomai Biotechnology Ltd. (Hefei, China). Graphene oxide (GO) solution was purchased from Nanjing XFNANO Materials Tech Co., Ltd. (Nanjing, China). Selection buffer (DPBS) was prepared with sterilization ultrapure water (137 mM NaCl, 2.7 mM KCl, 1.5 mM KH_2_PO_4_, 8 mM Na_2_HPO_4_, 1 mM CaCl_2_, 0.5 mM MgCl_2_, and 1 L, pH = 7.4). UNIQ-10 Spin Column Oligo DNA Purification Kit was purchased from Sangon Biotech Ltd. (Shanghai, China). All other analytical pure chemical reagents were purchased from Damao Chemical Reagent Factory (Tianjin, China) and Sigma-Aldrich (Shanghai, China). All solutions were treated with sterilization ultrapure water.

### 2.2. Capture-SELEX Selection for MG ssDNA Aptamer

#### 2.2.1. ssDNA Library Immobilization

The procedure of Capture-SELEX for aptamers against MG was shown in Scheme 1a. In brief, in the first round, the synthetic initial ssDNA library at 1.3 nmol was dissolved and diluted with DPBS to a final concentration of 5 μM and the primer Biotin-CO was mixed with the library at a ratio of 2:1. The library and capture oligo primers underwent a slow denaturation and renaturation (95 °C for 10 min, 60 °C for 1 min, and 25 °C for 1 min, 0.1 °C/s), which was closely followed by a mixture with the streptavidin magnetic beads and incubated with rotation at room temperature for 40 min to immobilize ssDNA library. The slow denaturation and renaturation was performed using PCR instrument T100 Thermal Cycler (Bio-Rad, Hercules, CA, USA). After the incubation, the beads were washed with the selection buffer DPBS five times to remove nonspecifically adhered and dissociative cDNA probe. The concentrations of ssDNA library in the suspension before and after the incubation were monitored to calculate the library capturing efficiency using Nanodrop 1000 Spectrophotometer (Thermo Scientific, Wilmington, DE, USA). During the selection rounds, the negative selections were performed with selection buffer to remove the nonspecific sequence interference, followed by adding the 200 μL, 100 μM MG oxalate standard solution for 40 min positive selection. 

#### 2.2.2. Monitoring Selection Process 

The ssDNA libraries with known different concentrations were amplified in Q-PCR to obtain the quantitative standard curve between the concentration of ssDNA and Cq value for quantitative ssDNA recovered during screening. After the incubation, the beads and the supernatant were separated using a strong magnet. The suspension served as a template for Q-PCR to determine the recovery of bound ssDNA by absolute quantitative method [20]. The 2 μL template mixed with 30 μL Q-PCR mix for amplification under the reaction conditions that were 95 °C for 2 min, 95 °C for 0.5 min, 60 °C for 0.5 min, and 72 °C for 0.5 min with 25 cycles. The Q-PCR was performed using Roche LightCycler 96 (Roche, Basel, Switzerland).

#### 2.2.3. Positive ssDNA Amplification

The other eluent suspension from positive selection was mixed with the emulsion PCR mix (10 μL FAM-Forward at a concentration of 100 µM, 10 μL PolyA_20_-Reverse at a concentration of 100 µM, 200 µL 10× PCR buffer, 40 µL 1U high fidelity DNA polymerase (Toyobo Biotech Co. Ltd., Shanghai, China), 40 µL dNTP mix at a concentration of 10 mM, sterile water up to a total volume of 2 mL) for PCR amplification of bound ssDNA. By adding 8 mL emulsifier (Anhui Angputuomai Biotechnology Ltd., Hefei, China), the mixture was vortexed for 3 min and left to stand for 5 min. The PCR program was performed at the condition: 95 °C for 2 min, 95 °C for 1 min, 60 °C for 1 min, and 72 °C for 1 min with 25 cycles. After the amplification, n-butyl alcohol was used to concentrate the PCR product, followed by adding 2× TBE-urea buffer to boil for 10 min at 100 °C for degeneration. The FAM-ssDNAs were separated by 12% 7 M urea denaturing polyacrylamide gel electrophoresis (PAGE) that was performed at 300 V for 45 min by PowerPa Bole small electrophoresis instrument (Bio-Rad, Hercules, CA, USA) to verify and obtain the ssDNA [21,22]. The fluorescent strip was cut off and boiled to collect the secondary ssDNA library, which was purified and dialyzed overnight in a selection buffer.

The following selection process was similar to the first selection round, but the concentration of input ssDNA library was reduced to 800 nM (100 μL) and the volume of DPBS was reduced to 200 μL to accelerate screening pressure. After nine rounds of aptamer selection, the recovery rate of ssDNA no longer increased and the selection ended. Detail selection setting conditions are shown in Table S1.

### 2.3. High-Throughput Sequencing (HTS) and Sequence Analysis 

The enrich library at the ninth round and other rounds’ libraries were purified and prepared as the templates, which were amplified in the PCR mix containing different primer barcodes. The PCR product DNA was recovered using UNIQ-10 Spin Column Oligo DNA Purification Kit. Then, the samples were sent to Anhui Angputuomai Biotechnology Co., Ltd. (Hefei, China) for HTS at an Illumina PE150 HTS platform.

After sequencing, the reads of each round, especially the final one, were counted and clustered. Clustered sequences provided the basis for downstream analysis, such as alignment and sequence motif discovery [23]. According to homologous comparison and evolutionary tree analysis by Cluster X2 and MEGA-X software, the enriched sequences were picked out for prediction of free energy (ΔG) and secondary structure at online bioinformatics platforms Mfold (http://www.unafold.org/, accessed on 10 March 2022).

### 2.4. Affinity Analysis of the Candidate Aptamers by ITC

Finally, enriched sequences identified through HTS and sequence analysis were individually synthesized and tested for binding affinity and specificity. Isothermal titration calorimeter (ITC) studies were performed to determine the dissociation constants (K_d_) to evaluate the affinity of the candidate aptamers using a MicroCal ITC200 isothermal titration calorimeter (Microcal Inc., Northampton, MA, USA) at 25 °C. The Origin scientific software version 7 (Microcal Software Inc., Northampton, MA, USA) was used to read and analyze the experimental data. The candidate aptamers were dissolved and filtered using a 0.45 μm membrane filter in DPBS to a final concentration of 5 μM, which filled the ITC cell. The syringe was filled with 2 mM of MG oxalate standard solution in the same buffer. A total of 20 injections of MG were added to the ITC cell with an initial 0.4 μL injection having 60 s initial equilibrium delays. The samples were stirred at a speed of 1000 rpm throughout the experiment, and thermal titration data were fitted to the one-site binding model to determine the K_d_ values.

### 2.5. General Procedure of GO-Based Fluorescence Assay to Detect MG

The sensitivity of determination of GO-based fluorescence assay for MG oxalate detection by aptamers was carried out under the optimized condition. The found aptamers were modified with FAM at the 5′ end. For MG oxalate detection, the total reaction system to 700 μL was carried out in a 1.5 mL plastic vial. 5′-FAM-MG-aptamers were initially dissolved and diluted with DPBS to a final concentration of 10 nM. Then GO was added and mixed gently at an optimized concentration to absorb 5′-FAM-aptamers and quench the fluorescence at room temperature. After 10 min incubation, a series of different final concentrations of MG oxalate standard solutions (0, 4, 8, 16, 30, 60, 100, 150, 200, 300, 400, 550, 650, 750, 800, 1000, 1200, 1500, 1800, 2000, 3000, 4500, 6000, 10,000, 15,000, 20,000 ng/mL) were separately added and mixed well for 20 min to form the MG-aptamer complex at room temperature, resulting in the desorption of FAM-aptamers from GO. MG oxalate standards were prepared with ultrapure water. Next, 700 μL of the resulting solution was transferred to an average of 200 μL each to the blackboard wells, and then the fluorescence intensity of the biosensing system was read and recorded to measure the effects of MG oxalate on the fluorescence recovery degree. Fluorescent intensity was recorded under 490 nm excitation and 520 nm emission using the Biotek SYNERGY H1 microplate reader (Gene Company Limited, Chai Wan, Hong Kong, China). Each reaction was repeated and tested 3 times.

The specificity and selectively tests of the developed GO-based fluorescence assay were also determined using the 5′-FAM-MG-aptamer. The procedures were performed under the same condition, while the similar veterinary drugs SEM, AOZ, AHD, AMOZ, CAP, LMG, SMZ, CV, and SP standard solutions were prepared at two times the concentration of MG for the specificity test. The fluorescence intensity of each reaction system was also recorded for comparison.

### 2.6. Determination of MG in Actual Water Samples 

To verify the feasibility and reliability of applying an aptasensor method in practical applications, the actual water samples were collected from the aquatic product market. Before being spiked with different concentrations of MG standard samples (100, 1000, and 2000 ng/mL), simple pretreatments, such as standing, filtering by 0.22-μm film, and diluting 5-fold with DPBS, were carried out on the actual water samples to remove solid impurities. In addition, the fluorescence intensity of reaction samples was measured and analyzed.

## 3. Results and Discussion

### 3.1. Capture-SELEX Selection of Aptamers for MG

**Figure 1:**
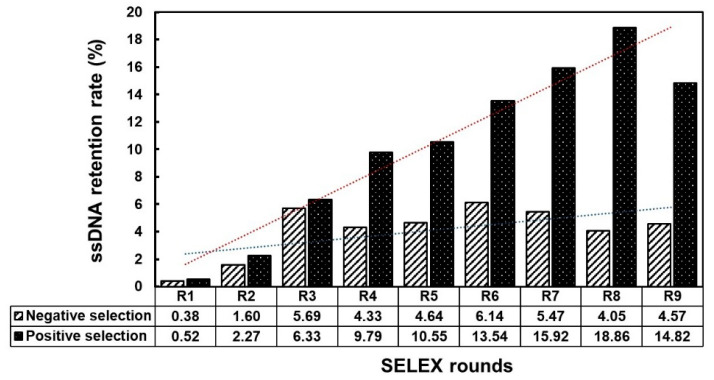
ssDNA retention rate of the selected ssDNA in the solution in each round of selection.

Nine rounds of Capture-SELEX were performed for successful enrichment of the ssDNA which have possible affinity target MG (Scheme 1a). The details of the selection condition are shown in Table S1. During the selection, Q-PCR absolute quantitative method was carried out to monitor the selection process according to the Cq values and amplification curve. The quantitative standard curve, which was made by the known different concentrations of ssDNA and Cq value that was obtained from the amplification, is shown in Figure S1. The standard curve correlation coefficient R^2^ was 0.9948 (y = −0.2335x + 5.0203, where the y was the logarithm DNA concentration and x was the Ct value), showing a good linear correlation between Cq value and ssDNA concentration. After the positive incubation, the amount of ssDNA target to MG was calculated based on the Cq value. With the increase in selection rounds, the retention rate of ssDNA in the positive selection increased from 0.52% to 18.86%. The retention rates of ssDNA were compared between positive and negative selection to judge the screening process. The ratio of the retention rate of the positive screen to that of the negative screen indicated that the screening was more effective. The retention rate increased to a maximum of 4.657 at round 8 then began to decrease. In round 9, the retention rate of positive selection began to decrease to 14.82, indicating that the ssDNA library was no longer enriched and the SELEX ended (Figure 1).

### 3.2. High-Throughput Sequencing and Sequence Analysis of the Enriched Library

Every ssDNA library of selection round was purified and sequenced using the high-throughput sequencing on the Illumina platform. Especially, the sequences observed in the ninth enriched round were sorted by frequency of occurrence. The first 100 sequences in order of number were selected for sequence analysis. To compare the difference of sequences, the 40 nt random sequence fragment underwent homologous alignment and phylogenetic tree analysis. The Clustal X2 software labeled the same bases in the sequence with the same color for identification. The phylogenetic tree analysis classified sequences by similarity to indicated differences between sequences. The sequences of different branches from the same tree root may be responsible for a similar function. Not only that, the prediction of secondary structure and the low free energy of sequences were performed using a web-based tool Mfold, which provided a reference for discovering the structural motifs. Overall, 24 candidate sequences (Table S2) were selected and synthesized for the following affinity and specificity experimental analysis. 

### 3.3. K_d_ Values of Active Aptamers

**Figure 2:**
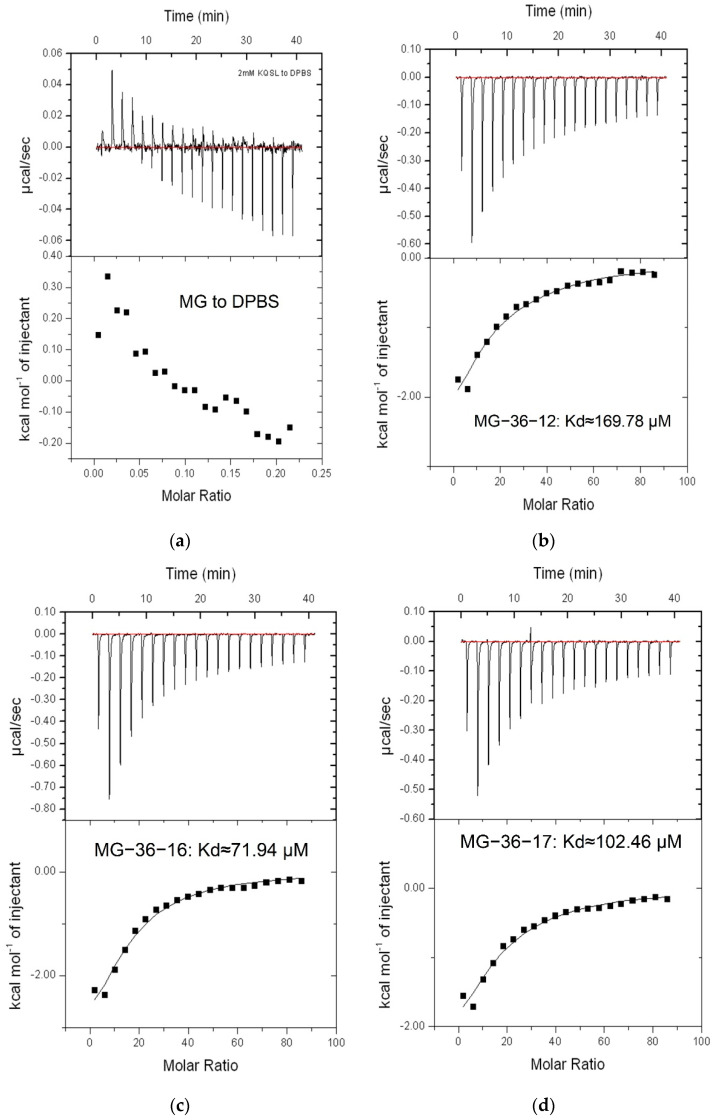
ITC analysis of binding dissociation constants (K_d_). (**a**) 2 mM MG oxalate standard solution to DPBS. (**b**–**d**) The saturation curves and K_d_ of aptamer MG-36-12, MG-36-16, and MG-36-17 were obtained by ITC analysis.

**Figure 3:**
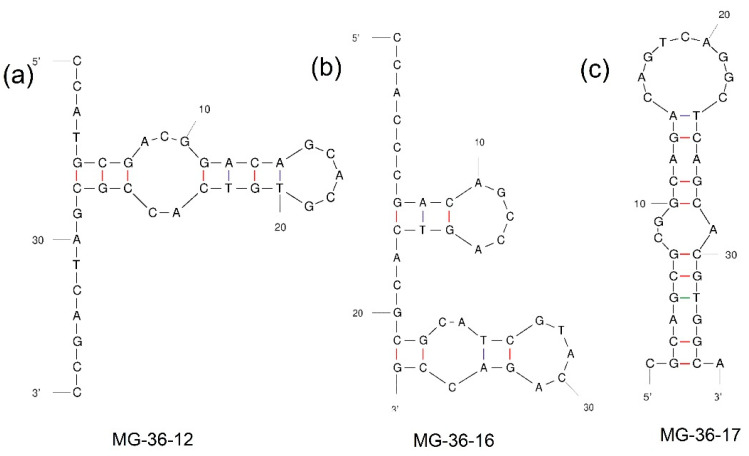
The secondary structures of the MG-36-12 (**a**), MG-36-16 (**b**), and MG-36-17 (**c**) candidate aptamer were predicted by Mfold.

Totally, 24 aptamer candidates were synthesized and diluted by DPBS, and then they were subjected to ITC for determining their binding affinity toward the analyte (MG in this case). Under the experiment condition of 100 μM aptamer candidates and 10 mM MG, 21 aptamer candidates were eliminated owing to the weak thermal variation in the reaction. MG-36-12/16/17 aptamers showed apparent thermal variation with the constant injections of MG. The number 36 represents the number of nucleotides and 12/16/17 represents the number of different aptamers. Three independent assays were performed to optimize the binding conditions. Two hundred microliters of 2 mM MG was titrated into three hundred microliters of 5 μM MG-36-12/16/17 aptamers in the cell at 25 °C. Based on the one-site model to create a fitted curve, the ITC results show that the K_d_ of MG-36-12/16/17 aptamers were estimated to be 169.78 μM, 71.94 μM, and 102.46 μM, respectively (Figure 2). Secondary structure models of these three aptamers were predicated showing the fact that two or three stem-loop features of aptamers may be formed to bind with the analyte (Figure 3). The predicted folding free energy of MG-36-12/16/17 aptamers were −5.87 kcal/mol, −3.94 kcal/mol, and −4.55 kcal/mol, respectively (Table 2). Among three aptamers, the MG-36-16 aptamer seemed to present the strongest binding affinity for MG oxalate because of the lowest K_d_ value. To verify the ability of each aptamer to detect MG in the aptamer-based GO-fluorescence sensor, three sequences were used in the following aptasensor development and specificity test.

### 3.4. Development and Optimized Conditions of GO-Based Fluorescent Aptasensor

#### 3.4.1. Development of the GO-Based Fluorescent Aptasensor for Detection of MG

A sensitive GO-based fluorescent aptasensor for MG detection was developed based on the MG-induced fluorescence intensity signal change. A schematic of the sensing principle is shown in Scheme 1b. Firstly, the dye-labeled ssDNA probes: 5′-FAM-MG-36-12/16/17 aptamers were absorbed on the surface of the GO owing to the strong π–π stacking effect between the nucleobases of aptamer and the sp^2^ atoms of GO, accompanied by the decrease in fluorescent intensities due to FRET [17,24]. Next, in the presence of analyte MG, the aptamer folded into a special structure for preferentially binding to the MG analyte owing to a strong affinity effect, which leads to the separation of aptamers from GO. As reported previously, the aptamer–target compound was powered and maintained by hydrophobic forces, electrostatic forces, and hydrogen bond interactions. Therefore, the fluorescence emission of aptamers was recovered, for it obstructed the energy transfer between the FAM and GO. The fluorescence intensity of the FAM-labeled aptamer was monitored and calculated to analyze the quantity of MG. A fluorescence aptasensor model was established to detect the MG and the other similar contaminates for specificity test. All the fluorescence signals were collected by microplate reader at 490 nm and 520 nm.

#### 3.4.2. Optimization of Detection Conditions

**Figure 4:**
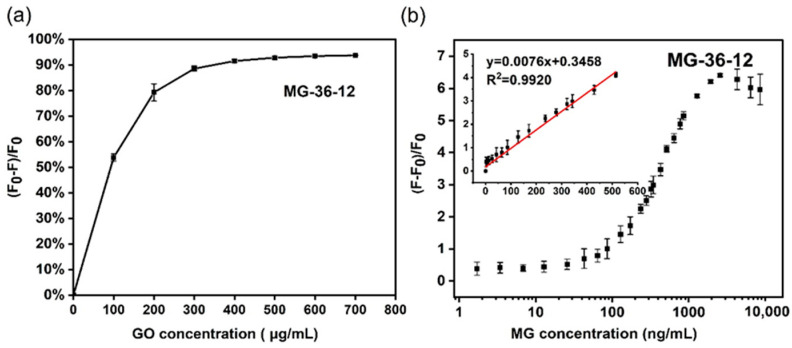
GO concentration optimization and sensitivity test of aptamer MG-36-12 in GO-based fluorescent aptasensor. (**a**) GO concentration for the fluorescence quenching of FAM-labeled aptamer, MG-36-12. (**b**) The relative fluorescence intensity of FAM aptamer after incubation with a series of concentrations of MG (0–8571.43 ng/mL). The inset shows a linear relationship (R^2^ = 0.9920) with the concentration of MG in the range of 1.71–514.29 ng/mL. Error bars were obtained from three parallel experiments.

**Figure 5:**
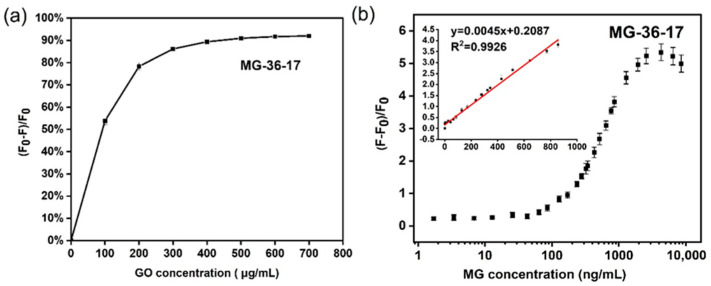
GO concentration optimization and sensitivity test of aptamer MG-36-17 in GO-based fluorescent aptasensor. (**a**) GO concentration for the fluorescence quenching of FAM-labeled aptamer MG-36-17. (**b**) The relative fluorescence intensity of FAM aptamer after incubation with a series of concentrations of MG (0–8571.43 ng/mL). The inset shows a good linear response from 1.71 to 857.14 ng/mL of MG (R^2^ = 0.9926). Error bars were obtained from three parallel experiments.

This fluorescent aptasensor depended on relevant experimental factors, including aptamer concentration, GO concentration, and reaction buffer. The FAM-MG-aptamer, with a final concentration at 10 nM, showed a proper fluorescence intensity in the solution. To explore the quenching efficiency of GO to FAM-labeled aptamer, a series of concentrations of GO from 0 to 600 μg/mL was added into a microcentrifuge tube containing 10 nM FAM-aptamer. For MG-36-12, the result in Figure 4a shows that 300 μg/mL GO was suitable for quenching the fluorescence intensity to approximately 90%, and the fluorescence intensity did not decrease along with the increase in GO concentration. For MG-36-17, the result in Figure 5a shows that 400 μg/mL GO was suitable for quenching the fluorescence intensity to approximately 90%. The quenching efficiency was expressed by (F_0_ − F)/F_0_, where F_0_ was the original fluorescence intensity of 10 nM FAM-aptamer without GO, and F was fluorescence intensity with an addition of different concentrations of GO. Both were collected by microplate reader at 490 nm and 520 nm. 

To obtain a better response of fluorescent signal, the reaction buffer was DPBS (pH 7.4), which was used in the aptamer screening procedure. For better sensing efficiency, the suitable time for the absorption of FAM-aptamer onto GO was 10 min, and the conjugation of FAM-aptamer to MG was 20 min at room temperature. With the increasing concentration of MG, the fluorescence intensity of FAM hardly changed to ensure that fluorescence intensity in the reaction was the fluorescence emission recovery caused by conformational folding of the FAM-labeled aptamer off GO (Figure S2).

#### 3.4.3. Sensitivity and Specificity Test

Under the mentioned optimized condition, the GO-based fluorescent aptasensor sensing method was established to detect the MG. In this aptasensor, a series of different concentrations of MG at 300 μL (0 to 20,000 ng/mL) were added to the aptamer FAM-MG-36-12/16/17 with the GO complex. The relative fluorescence intensity of the FAM-labeled aptamer/GO complex in the presence of MG was measured. The resulting calibration curve for MG detection was obtained by determining the relationship between the y (y = [F − F_0_]/F_0_) and the final concentration of MG (x, ng/mL). The equation y = (F − F_0_)/F_0_ was calculated to represent the relative fluorescence intensity, where F_0_ is the original fluorescence intensity after quenching the FAM aptamer fluorescence by GO without MG, and F is the fluorescence intensity of the mixture after the incubation of MG with the GO/FAM-aptamer mixture. With an increasing concentration of MG, the relative fluorescence intensity increased. In the reaction system of FAM-MG-36-12, the resulting calibration curve for MG detection displayed a good linear relationship in the range of 1.71–514.29 ng/mL with a linear equation y = 0.0076x + 0.3458 and a good linearity correlation coefficient R^2^ = 0.9920 (Figure 4b). In a statistical process, the limit of detection (LOD) and limit of quantitation (LOQ) parameters were typically defined and expressed as LOD = 3σ/S and LOQ = 10σ/S, where σ is the standard deviation for blank samples, and S is the slope of the standard curve [25,26,27]. The LOD and LOQ of the aptasensor were estimated to be 0.79 ng/mL (1.70 nmol/L) and 2.63 ng/mL (5.68 nmol/L), respectively. Likewise, the good linear response, achieved by applying FAM-MG-36-17 in the detection strategy, was from 1.71 to 857.14 ng/mL of MG, with a linear equation y = 0.0045x + 0.2087 and a good linearity correlation coefficient: R^2^ = 0.9926 (Figure 5b). A statistical analysis revealed that the LOD and LOQ of this aptasensor, which applied aptamer MG-36-17, were 2.13 ng/mL (4.60 nmol/L) and 7.11 ng/mL (15.34 nmol/L), respectively. A significant positive correlation was found between the relative fluorescence intensity and MG concentration. Both aptamers MG-36-12 and MG-36-17 were demonstrated to greatly bind to MG in the aptasensor. The two LOD values were low enough to satisfy the requirements for trace MG detection. Compared with others’ work on the detection of MG, our method first used the DNA aptamers of MG oxalate as the stable identification molecule in a fluorescent biosensor. For example, Zhao and co-workers reported a colorimetric aptasensor using RNA MG aptamer to detect MG in a concentration range from 10 to 500 nmol/L with a LOD of 1.8 nmol/L [28]. Another colorimetric aptasensor based on RNA and gold nanoparticles was developed to detect MG with a LOD of 15.95 nM by Jia, etc. [29]. Although the LOD of our method was slightly higher, it was sufficient to meet the detection limit of MG in some countries. In Germany, MG was banned to be used as an animal drug because of the possible carcinogenic, mutagenic, and teratogenic risks for human health. A zero tolerance of 0.01 mg/kg for the sum of MG and LMG was established [1]. In the EU, the experts recommend a limit of up to 2 μg/kg for exposure to food contaminated with MG. Consequently, the GO-based fluorescent aptasensor was developed to detect MG using DNA aptamer, and the results show its feasibility, sensitivity, competitive detection limit, and wider detection linear range.

Additionally, aptamer MG-36-16 performed relatively weakly in detecting MG in the aptamer-based fluorescence sensor compared with the above-mentioned two aptamers, as shown by the fluorescence signal instability and weak fluorescence emission (Figure S3). In this GO-based fluorescent aptasensor, we conjectured that the FAM modification weakened the ability of MG-36-16 to recognize MG in this aptamer-based FRET assay [30,31]. Therefore, we lengthened both ends of MG-36-16 by adding primer sequences (each primer contains 20 nucleotides) and polyA_6_ at the 5′ end to obtain aptamer MG-76-16-6A, resulting in a FAM label modification away from the MG-36-16 core sequence. The results reveal that our attempt was effective in enhancing the detection ability of MG-36-16 on MG, but its effect could still not be compared with those of MG-36-12 and MG-36-17 (Figure S4a). Aptamer MG-76-16-6A had a correlation linearity in a range from 1.71 to 321.43 ng/mL (R^2^ = 0.9572).

Aptamer MG-76-16-6A: 5′-AAAAAA-GTTCGTGGTGTGCTGGATGT-CCACCCGACAGCCAGTCACGCGCATCGTACAGACCG-TGACACATCCAGCAGCACGA-3′.

**Figure 6:**
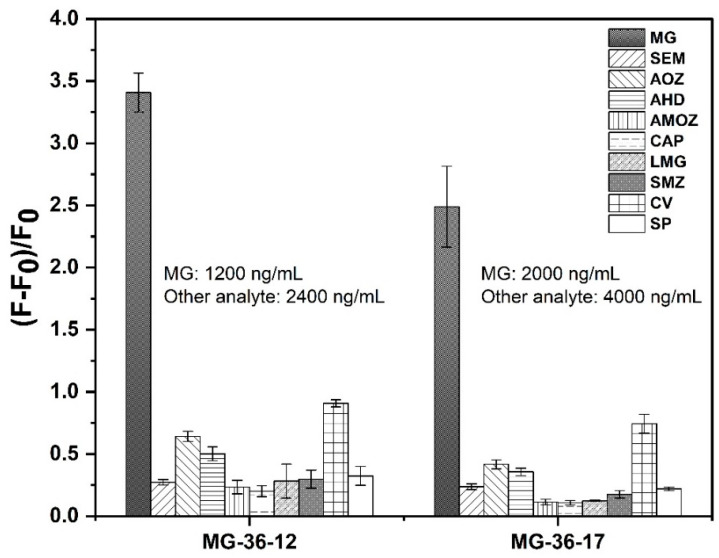
Fluorescence results were obtained in the presence of MG and the different analytes samples. The ratio of (F − F_0_)/F_0_ was calculated to show the relative fluorescence intensity of aptamer.

Verification of the specificity of MG-36-12, MG-36-17 (Figure 6), and MG-76-16-6A (Figure S4b) to MG was measured in the fluorescence assay. We compared the relative fluorescence intensity in the presence of MG and other relevant veterinary drugs including SEM, AOZ, AHD, AMOZ, CAP, LMG, SMZ, CV, and SP in the test, where the concentration of other targets was twice that of MG, all used at the same optimal reaction conditions. The result indicates that three aptamers showed high and comparable specificity in differentiating MG, with the highest relative fluorescence intensity from such several analytes that had no effective binding to the aptamer. Moreover, at the lower concentration of MG, MG-36-12 was applied to obtain a higher relative fluorescence signal change close to 3.5 than MG-36-17. Aptamer MG-36-12 performed well because of its sensitivity and specificity to MG. These results demonstrate that two DNA aptamers with not only high affinity but also high specificity could be applied to detect MG contaminants in the GO-based fluorescent aptasensor for potential application in MG detection.

### 3.5. Practicability for Determination of MG in Actual Water Samples

The disinfection of the transport and temporary ponds with MG and the addition of a certain amount of MG to the water can significantly reduce the mortality of fish. For food safety and environmental protection, sensitive, effective, and user-friendly methods to detect food contaminants were required to provide an instant detection [32]. Detection assays, where aptamers were used for biosensors in real samples, are still rare and a pretreatment protocol and matrix dilution are usually required before aptamer application. Before the test, a filtration by a 0.22 μm film and 5-fold dilution of a water sample from the aquatic product market with DPBS could remove impurities and maintain the aptamer’s ability to recognize MG in a suitable buffer system, which was the same as the SELEX reaction buffer [33]. The pretreated sample solutions were added to three different concentrations of MG standard solution. The results are summarized in Table 3, showing that the recovery rates were evaluated between 81.54% and 100.96% based on triplicate experiments at each concentration. These results prove the feasible practicability of our MG aptamer and the fluorescent aptasensor for the determination of MG in actual water. As demonstrated in Table 3, these results indicate that this aptasensor has great potential for practical applications.

## 4. Conclusions

In conclusion, we obtained three different and specific MG oxalate aptamers that came from DNA library-immobilized Capture-SELEX technology through nine rounds of selection. This method immobilized the library onto magnetic beads instead of MG oxalate, ensuring that the exposure of the intact construct of targets and the target-induced aptamer structure change, which further benefits the aptamer development of small molecules. After the performance test of these three aptamers, the K_d_ values were obtained by ITC assays at 169.78, 71.94, and 102.46 μM to MG-36-12, MG-36-16, and MG-36-17, respectively. Furthermore, a simple and highly sensitive fluorescent aptasensor was developed for the quantitative, sensitive, and specific detection of MG. Aptamer MG-36-12 and MG-36-17 performed well in our fluorescent aptasensor, due to the sensitivity with LOD of 0.79 ng/mL and 2.13 ng/mL, respectively. Aptamer MG-36-16 was lengthened by adding the primer sequences and polyA to avoid possible effects due to FAM label modification, which led to restoring its ability to recognize MG in fluorescent aptasensors. In addition, MG aptamer was successfully applied for MG detection in real water samples from the aquatic product market, in which some impurities coexisted in a complex system in this fluorescent aptasensor. This fluorescent aptasensor had the advantages of high sensitivity and satisfactory recovery in detecting MG in real samples, revealing its application prospects in food safety and environmental monitoring.

## Figures and Tables

**Table 1:** Primer sequences used in SELEX.

Primer	Sequences (5′–3′)
Biotin-CO	5′-biotin-TCGTGCTGCTGGATGTGTCA-3′
FAM-Forward	5′-FAM-GTTCGTGGTGTGCTGGATGT-3′
PolyA_20_-Reverse	5′-AAAAAAAAAAAAAAAAAAAAAAAAA-Spacer18-TCGTGCTGCTGGATGTGTCA-3′
Q-Forward	5′-GTTCGTGGTGTGCTGGATGT-3′
Q-Reverse	5′-TCGTGCTGCTGGATGTGTCA-3′

**Table 2:** Sequence (5′-3′) predicted folding energy (ΔG) and dissociation constants (K_d_) values of aptamer candidates.

Aptamer	Sequence (5′–3′)	ΔG (kcal/mol)	K_d_ (μM)
MG-36-12	CCATGCGACGGACAGCACGTGTCACCGCGATCAGCC	−5.87	169.78
MG-36-16	CCACCCGACAGCCAGTCACGCGCATCGTACAGACCG	−3.94	71.94
MG-36-17	CGCAGCGCGGCAGACAGTCAGGCTCAGCACGTGGCA	−4.55	102.46

**Table 3:** Detection of MG in 5-fold diluted actual water from the aquatic product market using aptamer MG-36-17 in GO-based fluorescent aptasensor.

Sample	Add MG Concentration (ng/mL)	Final Concentration of MG (ng/mL)	GO-Based Fluorescent Aptasensor (ng/mL)	Recovery Ratio %	RSD (%)
1	100	42.86	37.73	88.03%	14.69%
2	1000	428.57	432.68	100.96%	3.24%
3	2000	857.14	698.92	81.54%	6.41%

## Data Availability

Data is contained within the article.

## Supplementary Materials

The following supporting information can be downloaded at: https://www.mdpi.com/2304-8158/11/6/801/s1, Table S1. Detail selection setting conditions in each round of Capture-SELEX; Table S2. 24 candidate sequences; Figure S1. The quantitative standard curve between DNA concentration and Cq of Q-PCR. Figure S2. Influence of high MG concentration factor on the fluorescence intensity of FAM labels in the sensing system. Figure S3. The fluorescence emission recovery of using FAM-MG-36-16 aptamer in the aptasensot after incubation with a series of concentrations of MG; Figure S4. (a) The relative fluorescence intensity of FAM-MG-76-16A aptamer after incubation with various concentrations of MG (0−1928.57 ng/mL). The inset showed a linear response from 1.71 to 321.43 ng/mL of MG (R^2^ = 0.9572). (b) The specificity test using aptamer MG-76-16-6A in aptasensor. Error bars were obtained from 3 parallel experiments.
