# Correction: Xie et al. Selection and Application of ssDNA Aptamers for Fluorescence Biosensing Detection of Malachite Green. *Foods* 2022, *11*, 801

**DOI:** 10.3390/foods11131933

**Published:** 2022-06-29

**Authors:** Miaojia Xie, Zanlin Chen, Fengguang Zhao, Ying Lin, Suiping Zheng, Shuangyan Han

**Affiliations:** 1 Guangdong Key Laboratory of Fermentation and Enzyme Engineering, School of Biology and Biological Engineering, South China University of Technology, Guangzhou 510006, China; 201921046785@mail.scut.edu.cn (M.X.); 202121050086@mail.scut.edu.cn (Z.C.); feylin@scut.edu.cn (Y.L.); spzheng@scut.edu.cn (S.Z.); 2 School of Light Industry and Engineering, South China University of Technology, Guangzhou 510006, China; fgzhao@scut.edu.cn

## Errors in Figure and Table

In the original publication [1], there was a mistake in Figure 4, Figure 5 and Table 3 as published. 

In Section “2.5. General Procedure of GO-Based Fluorescence Assay to Detect MG”, we described adding a series of different final concentrations of MG oxalate standard solutions (0, 4, 8, 16, 30, 60, 100, 150, 200, 300, 400, 550, 650, 750, 800, 1000, 1200, 1500, 1800, 2000, 3000, 4500, 6000, 10,000, 15,000, 20,000 ng/mL) into the reaction systems.

However, we found that these concentrations of MG were in fact not the final concentrations. In reality, we added a range of MG concentrations at a volume of 300 µL to the reaction system for a total capacity of 700 µL, so that the MG concentrations were diluted.

Additionally, the correct final concentrations of MG were used as the horizontal coordinate in Figure 4b and Figure 5b, and thus changed the slope of the fitted curve, along with the calculation of LOD and LOQ because of the revised slope. In the statistical process, limit of detection (LOD) and limit of quantitation (LOQ) parameters were typically defined and expressed as LOD = 3σ/S and LOQ = 10σ/S, where σ was the standard deviation for blank samples, and S was the slope of the standard curve. So, the LOD and LOQ of this method need to be revised.

The corrected Figure 4b and Figure 5b, the legend of Figure 4 and Figure 5, and the revised LOD and LOQ appear below.

**Figure 4:**
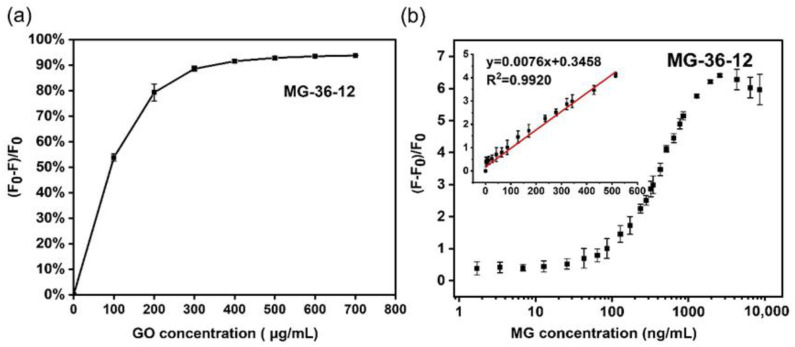
GO concentration optimization and sensitivity test of aptamer MG-36-12 in GO-based fluorescent aptasensor. (**a**) GO concentration for the fluorescence quenching of FAM-labeled aptamer, MG-36-12. (**b**) The relative fluorescence intensity of FAM aptamer after incubation with a series of concentrations of MG (0–8571.43 ng/mL). The inset shows a linear relationship (R^2^ = 0.9920) with the concentration of MG in the range of 1.71–514.29 ng/mL. Error bars were obtained from three parallel experiments.

**Figure 5:**
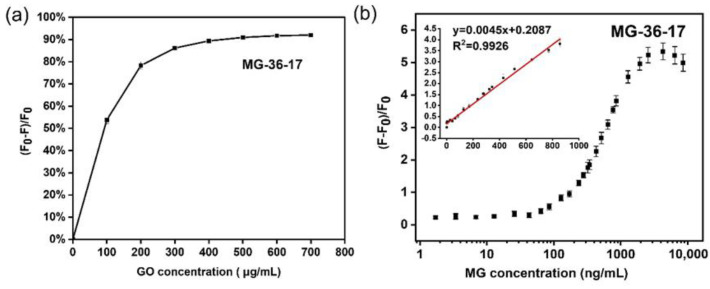
GO concentration optimization and sensitivity test of aptamer MG-36-17 in GO-based fluorescent aptasensor. (**a**) GO concentration for the fluorescence quenching of FAM-labeled aptamer MG-36-17. (**b**) The relative fluorescence intensity of FAM aptamer after incubation with a series of concentrations of MG (0–8571.43 ng/mL). The inset shows a good linear response from 1.71 to 857.14 ng/mL of MG (R^2^ = 0.9926). Error bars were obtained from three parallel experiments.

**Table 3:** Detection of MG in 5-fold diluted actual water from the aquatic product market using aptamer MG-36-17 in GO-based fluorescent aptasensor.

Sample	Add MG Concentration (ng/mL)	Final Concentration of MG (ng/mL)	GO-Based Fluorescent Aptasensor (ng/mL)	Recovery Ratio %	RSD (%)
1	100	42.86	37.73	88.03%	14.69%
2	1000	428.57	432.68	100.96%	3.24%
3	2000	857.14	698.92	81.54%	6.41%

## Correct Legend

1. Figure 4b was corrected, and the slope of fitted curve in Figure 4b was revised to 0.0076.
2. Figure 5b was corrected, and the slope of fitted curve in Figure 5b was revised to 0.0045.

## Text Correction

There were errors in the original publication.

“Since the final concentrations of the MG became smaller, abstract and some of the analyses of LOD and LOQ in the text need to be revised because of the corrected slopes of the fitted curves in Figure 4b and Figure 5b”.

Corrections were made to “Abstract”, “3.4.3. Sensitivity and Specificity Test”, “3.5. Practicability for Determination of MG in Actual Water Samples”, “4. Conclusions”. Errors in Abstract, Sections 3.4.3, 3.5, and the Conclusions section were corrected according to the new correct Figure 4b and Figure 5b. 

**Abstract:** Malachite green oxalate (MG) is a kind of veterinary drug, which is freely soluble in water and hazardous to aquatic products, resulting in food toxicity and human health problems. The demand for effective and sensitive detection of MG residues is increasing in food safety. In this work, three DNA aptamers MG-36-12/16/17 targeting MG with good affinity (K_d_ values were 169.78, 71.94, and 102.46 μM, respectively) were obtained by Capture-SELEX. Furthermore, MG-36-12, MG-76-16-6A, and MG-36-17 were found to perform sensitively and specifically to detect MG as a sensing probe in a FRET fluorescent aptasensor, where the FAM-labeled aptamer and GO were employed as efficient energy donor–acceptor pair. The linear range of this aptasensor using aptamer MG-36-12 was from **1.71 to 514.29** ng/mL and the LOD was as low as **0.79** ng/mL. Additionally, the fluorescent assay using aptamer MG-36-17 to detect MG exhibited a linear relationship from **1.71 to 857.14** ng/mL and a LOD of **2.13** ng/mL. Meanwhile, the aptasensor showed high specificity to MG with no cross-reactivity to other veterinary drugs and had a mean recovery of **81.54% to 100.96%** in actual water samples from the aquatic product market.

3.4.3. Sensitivity and Specificity Test

Under the mentioned optimized condition, the GO-based fluorescent aptasensor sensing method was established to detect the MG. In this aptasensor, a series of different concentrations of MG **at 300 μL** (0 to 20,000 ng/mL) were added to the aptamer FAM-MG-36-12/16/17 with the GO complex. The relative fluorescence intensity of the FAM-labeled aptamer/GO complex in the presence of MG was measured. The resulting calibration curve for MG detection was obtained by determining the relationship between the y (y = [F − F_0_]/F_0_) and **the final concentration of MG (x, ng/mL)**. The equation y = (F − F_0_)/F_0_ was calculated to represent the relative fluorescence intensity, where F_0_ is the original fluorescence intensity after quenching the FAM aptamer fluorescence by GO without MG, and F is the fluorescence intensity of the mixture after the incubation of MG with the GO/FAM-aptamer mixture. With an increasing concentration of MG, the relative fluorescence intensity increased. In the reaction system of FAM-MG-36-12, the resulting calibration curve for MG detection displayed a good linear relationship in the range of **1.71–514.29** ng/mL with a linear equation y = **0.0076**x + 0.3458 and a good linearity correlation coefficient R^2^ = 0.9920 (Figure 4b). In a statistical process, the limit of detection (LOD) and limit of quantitation (LOQ) parameters were typically defined and expressed as LOD = 3σ/S and LOQ = 10σ/S, where σ is the standard deviation for blank samples, and S is the slope of the standard curve [25–27]. The LOD and LOQ of the aptasensor were estimated to be **0.79** ng/mL (**1.70** nmol/L) and **2.63** ng/mL (**5.68** nmol/L), respectively. Likewise, the good linear response, achieved by applying FAM-MG-36-17 in the detection strategy, was from **1.71** to **857.14** ng/mL of MG, with a linear equation y = **0.0045x** + 0.2087 and a good linearity correlation coefficient: R^2^ = 0.9926 (Figure 5b). A statistical analysis revealed that the LOD and LOQ of this aptasensor, which applied aptamer MG-36-17, were **2.13** ng/mL (**4.60** nmol/L) and **7.11** ng/mL (**15.34** nmol/L), respectively.

Additionally, aptamer MG-36-16 performed relatively weakly in detecting MG in the aptamer-based fluorescence sensor compared with the above-mentioned two aptamers, as shown by the fluorescence signal instability and weak fluorescence emission (Figure S3). In this GO-based fluorescent aptasensor, we conjectured that the FAM modification weakened the ability of MG-36-16 to recognize MG in this aptamer-based FRET assay [30,31]. Therefore, we lengthened both ends of MG-36-16 by adding primer sequences (each primer contains 20 nucleotides) and polyA_6_ at the 5′ end to obtain aptamer MG-76-16-6A, resulting in a FAM label modification away from the MG-36-16 core sequence. The results reveal that our attempt was effective in enhancing the detection ability of MG-36-16 on MG, but its effect could still not be compared with those of MG-36-12 and MG-36-17 (Figure S4a). Aptamer MG-76-16-6A had a correlation linearity in a range from **1.71** to **321.43** ng/mL (R^2^ = 0.9572).

*3.5. Practicability for Determination of MG in Actual Water Samples*

The disinfection of the transport and temporary ponds with MG and the addition of a certain amount of MG to the water can significantly reduce the mortality of fish. For food safety and environmental protection, sensitive, effective, and user-friendly methods to detect food contaminants were required to provide an instant detection [32]. Detection assays, where aptamers were used for biosensors in real samples, are still rare and a pretreatment protocol and matrix dilution are usually required before aptamer application. Before the test, a filtration by a 0.22 μm film and 5-fold dilution of a water sample from the aquatic product market with DPBS could remove impurities and maintain the aptamer’s ability to recognize MG in a suitable buffer system, which was the same as the SELEX reaction buffer [33]. The pretreated sample solutions were added to three different concentrations of MG standard solution. The results are summarized in Table 3, showing that the recovery rates were evaluated between **81.54**% and **100.96**% based on triplicate experiments at each concentration. These results prove the feasible practicability of our MG aptamer and the fluorescent aptasensor for the determination of MG in actual water. As demonstrated in Table 3, these results indicate that this aptasensor has great potential for practical applications.

**4. Conclusions**

In conclusion, we obtained three different and specific MG oxalate aptamers that came from DNA library-immobilized Capture-SELEX technology through nine rounds of selection. This method immobilized the library onto magnetic beads instead of MG oxalate, ensuring that the exposure of the intact construct of targets and the target-induced aptamer structure change, which further benefits the aptamer development of small molecules. After the performance test of these three aptamers, the K_d_ values were obtained by ITC assays at 169.78, 71.94, and 102.46 μM to MG-36-12, MG-36-16, and MG-36-17, respectively. Furthermore, a simple and highly sensitive fluorescent aptasensor was developed for the quantitative, sensitive, and specific detection of MG. Aptamer MG-36-12 and MG-36-17 performed well in our fluorescent aptasensor, due to the sensitivity with LOD of **0.79** ng/mL and **2.13** ng/mL, respectively. Aptamer MG-36-16 was lengthened by adding the primer sequences and polyA to avoid possible effects due to FAM label modification, which led to restoring its ability to recognize MG in fluorescent aptasensors. In addition, MG aptamer was successfully applied for MG detection in real water samples from the aquatic product market, in which some impurities coexisted in a complex system in this fluorescent aptasensor. This fluorescent aptasensor had the advantages of high sensitivity and satisfactory recovery in detecting MG in real samples, revealing its application prospects in food safety and environmental monitoring.

## Wrong Citation

In the original publication, the wrong reference [14] after [12,13] in “the third paragraph” of “Introduction” was cited, which described that “**Sara, L.** selected an RNA aptamer…”. This should be revised to “**Grate, D.** selected an RNA aptamer of MG earlier, which was supposed to be less stable compared with DNA aptamer [14]”. 

The reference [14] needs to be replaced as below:

14. Grate, D.; Wilson, C. Laser-mediated, site-specific inactivation of RNA transcripts. *Proc. Natl. Acad. Sci. USA* **1999**, *96*, 6131–6136, doi:10.1073/pnas.96.11.6131.

The authors apologize for any inconvenience caused and state that the scientific conclusions are unaffected. This correction was approved by the Academic Editor. The original publication has also been updated.
