# Supplementary material for: Selection and Application of ssDNA Aptamers for Fluorescence Biosensing Detection of Malachite Green
Source: Foods. 2022 Mar 10;11(6):801. doi: 10.3390/foods11060801 (PMC8953565; doi:10.3390/foods11060801)
Supplement: Supplementary file 1 [file foods-11-00801-s001.zip › foods-1745531-supplementary.pdf]

Supplementary data

Selection and application of ssDNA aptamers for fluorescence biosensing detection of malachite green

Miaojia Xie[1], Zanlin Chen[1], Fengguang Zhao[2], Ying Lin[1], Suiping Zheng[1], Shuangyan Han[1*]

1 Guangdong Key Laboratory of Fermentation and Enzyme Engineering, School of Biology and Biological Engineering, South China University of Technology, Guangzhou, 510006, Guangdong, China

2 School of Light Industry and Engineering, South China University of Technology, Guangzhou, 510006, Guangdong, China

Table S1. Detail selection setting conditions in each round of Capture-SELEX

Round	The input of ssDNA library	Beads	Negative screening （40min）	Positive screening（40min）
1	260 µL/5 µM	1 mL	400 µL DPBS	200 µL/100 µM MG
2	100 µL/800 nM	80 µL	200 µL DPBS	200 µL/100 µM MG
3	100 µL/800 nM	80 µL	200 µL DPBS	200 µL/100 µM MG
4	100 µL/800 nM	80 µL	200 µL DPBS	200 µL/100 µM MG
5	100 µL/800 nM	80 µL	200 µL DPBS	200 µL/100 µM MG
6	100 µL/800 nM	80 µL	200 µL DPBS	200 µL/100 µM MG
7	100 µL/800 nM	80 µL	200 µL DPBS	200 µL/100 µM MG
8	100 µL/800 nM	80 µL	200 µL DPBS	200 µL/100 µM MG
9	100 µL/800 nM	80 µL	200 µL DPBS	200 µL/100 µM MG

Table S2. 24 candidate sequences. All 24 candidate sequences were selected from the enrichment library after the high-throughput sequencing and analysis of numerous sequences. The affinity of these 24 candidate sequences with MG was tested by ITC assays. Among them, MG-12, MG-16 and MG-17 labeled red were found to have an affinity for MG. The other sequences showed no affinity for MG.

Number	Sequence (5'-3')
MG-1	CCGCCACAGGACAGCCAGTCTGATCGCCATGACGAC
MG-2	AGCCCGAGCTGCAGCCAGAATGTGCCACGTGTACGT
MG-3	CGAGACACAGTCACGTGCATCCAGTCAGCATGCGTC
MG-4	TGCAACACAACAGCCAGTCATGCCGGTGCACAGTCA
MG-5	CCCCGACAGCCGGCACAATGTGTCTGCCACGGACGT
MG-6	CGCAACGCGGCAACAGAGCACAGATCGCACAGACTG
MG-7	CGAGCCGAACGAGACGTCCAGTCCGTGCCCACGCAC
MG-8	TGTGCCCACAACTCCAGTCACGTTCGTCCAGCTCAG
MG-9	TCTCGCAACAGCCAGCGTCATGTGACCACGGAACCA
MG-10	CGCGCAGCGGCAGACAGTCAGACTGCGACGCACGTT
MG-11	CATGCCCAACGACATGCAGCACGTGGCCATGCACAA
MG-12	CCATGCGACGGACAGCACGTGTCACCGCGATCAGCC
MG-13	ACGCATCGCGAACAGCCGTGCCGTGTCCACGTACAG
MG-14	CACCGCCAGGGACAGCCAGTCACGTAGCCCGGAACG
MG-15	CGGCCAGACGACAGCCAGTCACCGGACACGATCAGG
MG-16	CCACCCGACAGCCAGTCACGCGCATCGTACAGACCG
MG-17	CGCAGCGCGGCAGACAGTCAGGCTCAGCACGTGGCA
MG-18	CCGCAGCACGGTCGTACAGCAGCTGCCACGGCATCC
MG-19	CGCAGACAGCCAGTCAGGGAGCGTCCAGTCCGACAC
MG-20	CGCACAGCAAGACAGCCAGTCAACAGCCGCGAATGT
MG-21	CCATGCAGCCAGCGTCCACCGTCCGTACAGTACGTG
MG-22	CGCGCACAGACAGCGTCATGGCCACGTACAGTTCCT
MG-23	CGCTACGACAGACAGCGTCGAGCACACAACGTTGCG
MG-24	ACGCACGTACGGCCAGTCATATGCGCACCGCGTTCA

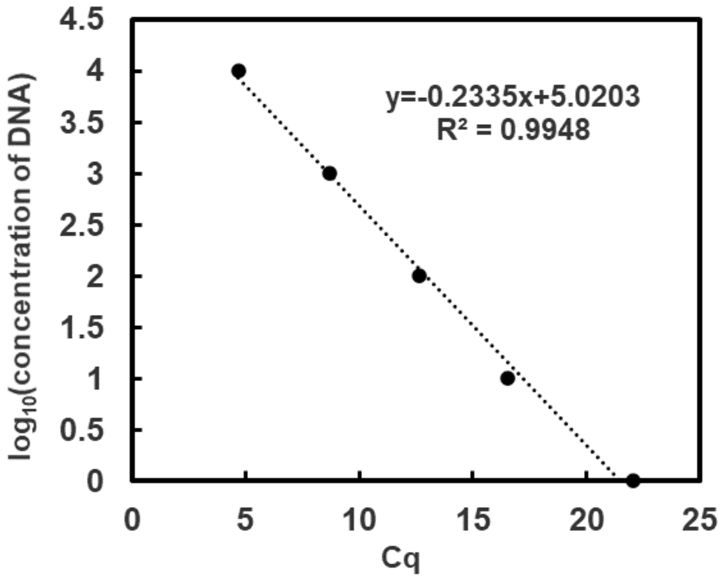

Figure S1. The quantitative standard curve between DNA concentration and Cq of Q-PCR.

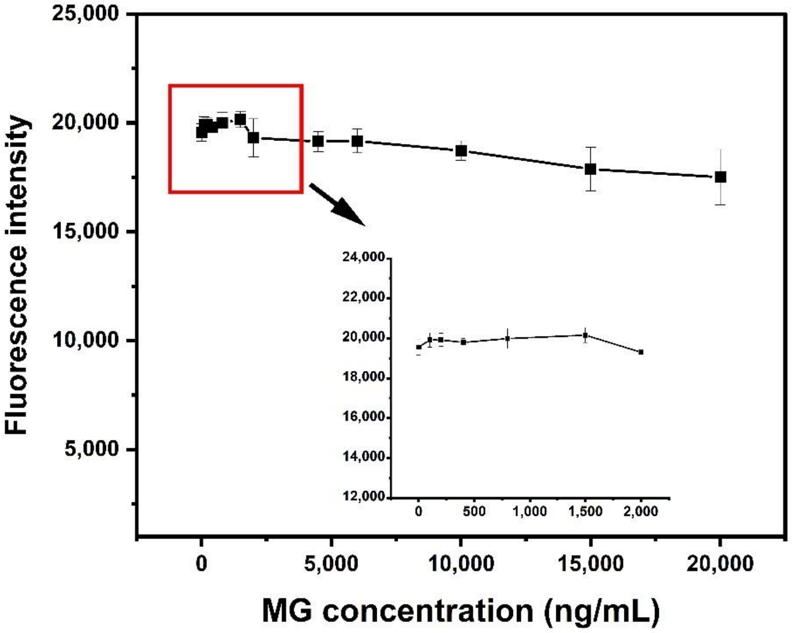

Figure S2. Influence of high MG concentration factor on the fluorescence intensity of FAM labels in the sensing system.

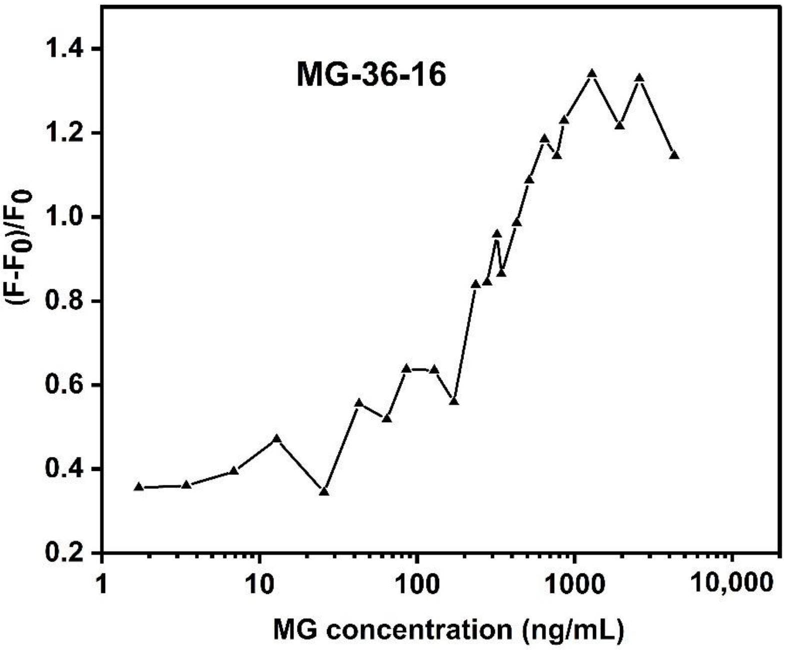

Figure S3. The relative fluorescence intensity of using FAM-MG-36-16 aptamer in the aptasensor after incubation with a series of concentrations of MG.

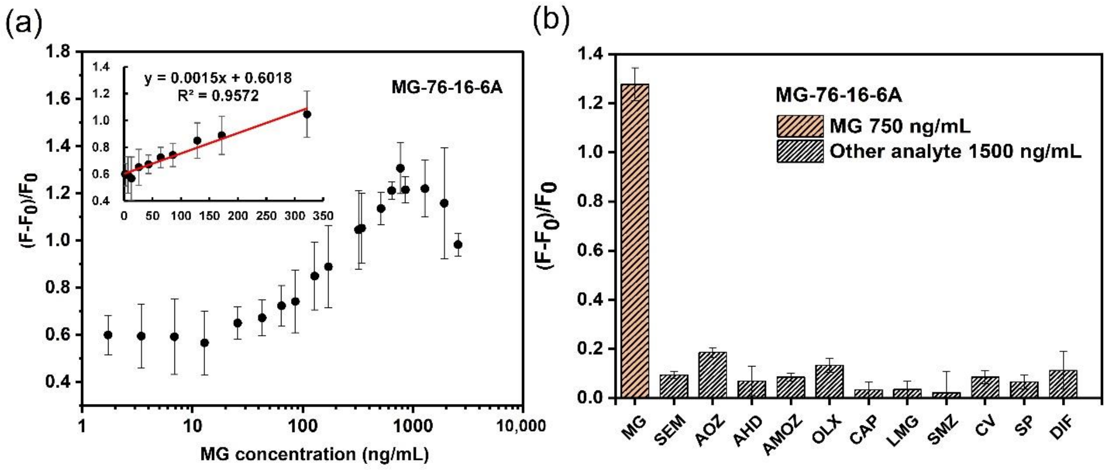

Figure S4. (**a**) The relative fluorescence intensity of FAM-MG-76-16-6A aptamer after incubation with various concentrations of MG (0-1928.57 ng/mL). The inset showed a linear response from 1.71 to 321.43 ng/mL of MG (R^2=0.9572). (**b**) The specificity test using aptamer MG-76-16-6A in aptasensor. Error bars were obtained from 3 parallel experiments.
